# Expression of caveolin-1 is correlated with disease stage and survival in lung adenocarcinomas

**DOI:** 10.3892/or.2011.1605

**Published:** 2011-12-21

**Authors:** PING ZHAN, XIAO-KUN SHEN, QIAN QIAN, QIN WANG, JI-PING ZHU, YU ZHANG, HAI-YAN XIE, CHUEN-HUA XU, KE-KE HAO, WEI HU, NING XIA, GUO-JUN LU, LI-KE YU

**Affiliations:** 1 First Department of Respiratory Medicine, Nanjing Chest Hospital, Nanjing 210029; 2 Department of Respiratory Medicine, Jinling Hospital, Nanjing University School of Medicine, Nanjing 210002; 3 Department of Respiratory Medicine, 81 Hospital of PLA, Nanjing 210002; 4 Department of Respiratory Medicine, Jiangsu Province Hospital of Traditional Chinese Medicine, Nanjing 210029, P.R. China

**Keywords:** caveolin-1, real-time PCR, immunohistochemical staining, non-small cell lung cancer, prognosis

## Abstract

Caveolin-1 (cav-1) has been implicated in the development of human cancers. However, the distribution of cav-1 in non-small cell lung cancer (NSCLC) and its significance require further study. Real-time PCR and Western blot assays were performed to detect cav-1 mRNA and protein levels in tumor tissues (TT) and matched tumor-free tissues (TF). The protein expression in 115 paraffin-embedded blocks was examined by immunohistochemical staining (IHC). Correlations between cav-1 mRNA and protein expression by IHC and clinicopathological features were statistically evaluated. For the 136 patients examined, the levels of cav-1 mRNA and protein expression were significantly lower in lung TT compared to matched TF (P<0.05). High cav-1 expression was detected in 60 of 115 (52.2%) NSCLC tissues and this level was significantly lower than cav-1 expression in non-cancerous lung tissues (15 of 19, 78.9%, P<0.05). Up-regulation of cav-1 mRNA expression in lung adenocarcinoma (AC) (29.7%) was higher than that observed in lung squamous cell carcinoma (SCC) (15.8%). Statistical analysis of the correlation between cav-1 protein expression and clinical features showed a statistical association with poorer N-stage (P=0.032) and higher pathological TNM stage (P=0.012) in lung AC patients, that was not found in lung SCC patients. Moreover, lung AC patients with higher cav-1 expression showed significantly shorter life-spans than those with lower cav-1 expression (P=0.032, log-rank test). The levels of cav-1 mRNA and protein expression were significantly lower in lung cancers when compared to matched TF or non-cancerous lung tissues. The higher protein expression correlated with the advanced pathological stage and shorter survival rates in lung AC patients.

## Introduction

Lung cancer remains the deadliest cancer worldwide despite improvements in diagnostic and therapeutic techniques (1). The reported incidences of lung cancer have yet to peak in many parts of world, particularly in China. The prognosis for lung cancer patients is generally poor, with an overall 5-year survival rate of approximately 10–15%, and it has shown little improvement in recent decades (2,3). Several independent prognostic factors for survival have been identified: performance status, disease stage, age, gender and the amount of weight loss (4). However, the discriminate value of most potential prognostic biologic markers is insufficient to predict the optimal therapeutic course for an individual. Thus, it is important to identify biological markers with predictive values for the survival of patients undergoing treatment.

Caveolin-1 (cav-1), a major structural component of specialized plasma membrane invaginations (caveolae), was first cloned in 1992 by Rothberg *et al* (5). There has been significant interest in understanding the structure and function of cav-1. The function of cav-1 in tumorigenesis remains controversial. In several *in vitro* studies, cav-1 was down-regulated in tumor cells isolated from the breast (6,7), cervix (8), lung (9,10) and ovary (11), and oncogenic transformation of cells was associated with a reduction in cav-1 expression (12,13). This evidence indicates that cav-1 may act as a tumor suppressor. Conversely, cav-1 is consistently up-regulated in bladder cancer (14), esophageal cancer (15) and prostate carcinomas (16), and this up-regulation has been associated with metastases and poor prognosis in prostate carcinoma and esophageal squamous cell carcinomas (SCC) (17,18). These results suggest that cav-1 can function as an oncogene rather than a tumor suppressor. Taking into consideration these conflicting *in vitro* and clinical results, we postulate that the functions of cav-1 may have different roles in various cancer cell types.

In primary lung cancer, only a few studies on cav-1 expression have been reported (19–24). In these studies, only immunohistochemical staining (IHC) was used to detect cav-1 expression. In SCC of the lung (20), the expression of cav-1 has been significantly correlated with the advanced pathological stage and poor prognosis. In a study by Kato, *et al* (22), cav-1 was found to serve as a tumor suppressor in lung adenocarcinoma (AC), with the loss of cav-1 regulation resulting in tumor extension and a lack of differentiation. In lung SCC, cav-1 overexpression may be correlated with tumor extension. However, the cav-1 mRNA/protein expression levels in lung tumor tissues (TT) compared to surrounding normal tissues and the association with clinicopathological factors in non-small cell lung cancer (NSCLC) is unknown.

In the present study, we have examined the cav-1 mRNA expression in 136 lung TT and matched tumor-free tissues (TF) using real-time PCR analysis and the protein expression in 20 paired lung TT and TF by Western blot analysis. In addition, the protein expression in another set of 115 paraffin-embedded blocks of NSCLC and 19 non-cancerous lung tissues was detected by IHC. The clinical significance of the cav-1 mRNA and protein expression levels detected by IHC with respect to the prognostic value of NSCLC patients is clarified.

## Materials and methods

### Patients and tissue specimens

During the period from August 2007 to May 2009, 136 lung cancer patients who received surgery at the Department of Thoracic Surgery of Nanjing Chest Hospital and the Jinling Hospital affiliated to the Nanjing University School of Medicine, were included in this study. TT and matched TF >5 cm distal from the tumor edge were immediately snap-frozen in liquid nitrogen and then stored at −80°C until use (protein and RNA isolation). Necrotic or hemorrhagic tissues were excluded. In addition, a set of 115 paraffin-embedded lung cancers and 19 non-cancer specimens between 2001 and 2003 were collected from the Nanjing Chest Hospital and the 81 Hospital of PLA. The 19 non-cancerous specimens included 5 pulmonary tuberculosis cases, 4 bronchiectasis cases, 6 lung bullae cases and 3 inflammatory pseudotumor cases. None of the patients received any chemotherapy or radiotherapy before surgery. Clinicopathological data were obtained by medical records in the archives room. The data included patient age, gender, smoking condition, tumor site, lymph nodal status and pathological stage. The postoperative pathological staging was determined according to the 7th edition of the TNM classification (25). Histological type was determined according to the classification by the World Health Organization. Inclusion criteria for this study were surgical complete resection of the tumor (resection margin microscopically free of tumor cells); patient survival for more than 3 months after surgery; and the patient did not die of causes other than lung cancer within 5 years following surgery. Follow-up information was obtained by phone investigations. The median follow-up of surviving patients at the time of analysis was 22 months (range, 3–81 months). The date of the last follow-up was March 21, 2009. The Research Ethics Committee of the Jinling Hospital affiliated to the Nanjing University School of Medicine approved this protocol and informed consent was obtained from all participants.

### RNA extraction and cDNA synthesis

Total RNA was isolated from frozen tissue with TRIzol (Invitrogen) using the manufacturer’s protocol. With random hexamer primers, 2 μg of RNA was reverse transcribed to cDNA using the PrimeScript™ first-strand cDNA synthesis kit (Takara), according to the manufacturer’s protocol.

### Real-time PCR

Primers for cav-1 and glyceraldehyde-3-phosphate dehydrogenase (GAPDH) were designed and synthesized by Sangon Biotech Co., Ltd. The primers were as follows: cav-1, forward, TTCGCCATTCTCTCTTTCCT, and reverse, CAGCTTCAAAGAGTGGGTCA; GAPDH; forward, GCACCGTCAAGGCTGAGAAC, and reverse, TGG TGAAGACGCCAGTGGA. Real-time PCR was performed in triplicate for each sample in a 20-μl reaction mixture, which consisted of template DNA (1 μl) and primers (0.2 μM), the ROX Reference Dye II (1X), dH_2_O (9.0 μl) and SYBR^®^ Premix Ex Taq (1X, SYBR Premix Ex Taq kit, Takara). PCR was performed on an ABI 7500 real-time PCR system using the following thermal settings: 1 cycle of 30 sec at 95°C, 45 cycles of 5 sec at 95°C and 34 sec at 60°C. The relative expression ratio (RR) of the cav-1 gene was calculated based on the Ct comparative method with the reference gene (GAPDH) in the sample.

### Western blot analysis

For protein analysis, samples were homogenized in lysis buffer (0.1% SDS, 50 mM Tris-HCl, pH 7.5, 1% NP-40, 150 mM NaCl, 1 mM Triton X-100, 1 mM EDTA) containing complete protease inhibitor (PMSF + P8340). Protein concentrations were measured by the BCA protein assay (Sigma). A total of 100 μg of protein was separated by 10–15% SDS-PAGE. The protein was then transferred to a nitrocellulose-membrane and these were saturated by incubating for 2 h with 5% non-fat dry milk in PBS/0.1% Tween-20 at 37°C. The membranes were incubated with the Mouse IgG monoclonal cav-1 antibody (BD Transduction Laboratories) overnight at 4°C. After 3 washes (5 min each) with PBS/0.1% Tween-20, membranes were incubated with the anti-rabbit immunoglobulin coupled to peroxidase (Abcam) at 37°C. After 1 h incubation, the membranes were washed 4 times (5 min each) with PBS/0.1% Tween-20 and the blots were developed using a chemiluminescence procedure (Amersham, Bioscence). The polyclonal anti-β-actin antibody served as the control.

### Immunohistochemical staining

Resected specimens were fixed in 10% formalin and paraffin-embedded blocks were prepared. Five millimeter sections were cut from the specimens and placed on slides coated with poly-L-lysine. IHC was performed using the EnVision two-step immunohistochemical method (EnVision Detection kit, Peroxidase/DAB, Rabbit/Mouse, Dako, Denmark), according to the manufacturer’s instructions. In brief, sections were routinely deparaffinized with xylene and rehydrated in decreasing concentrations of alcohol. Antigen retrieval was performed by placing the specimen in the EDTA retrieval agent at pH 8.0 and autoclaved at 12°C for 2 min to allow fixing. The sections were washed in phosphate-buffered saline (PBS) buffer (pH 7.6) and the sections were incubated overnight at 4°C in a moist chamber with the rabbit anti-human cav-1 polyclonal antibody (1:400, N-20: sc-894, Santa Cruz Biotechnology, Santa Cruz, USA). After washing the sections in PBS 3 times for 5 min the sections were treated for 30 min at room temperature in ChemMate EnVision+/HRP (Dako, Denmark). Subsequently, the sections were washed with PBS and diaminobenzidine (DAB) coloration was applied, followed by the application of a DAB solution (ChemMate EnVision+/DAB) until the color developed. Staining was monitored under bright-field microscopy and the reaction was stopped by washing with distilled water. The sections were then counterstained with hematoxylin, dehydrated in increasing concentrations of alcohol and coverslipped with neutral Gummi.

The slides were independently reviewed by 2 of the authors (P.Z. and X.-K.S.) who had no knowledge of the patients clinicopathological status. If discrepancies existed between the 2 reviewers, a consensus judgment was reached through discussion. The proportion of staining tumor cells in each selected field was determined by counting individual tumor cells at 4 randomly selected high magnification (x400) fields using light microscopy (Model CX31RTSF, Olympus, Tokyo, Japan). The immunoreactivities were graded as (−), (+), (++) and (+++) according to the percentage of positive tumor cells identified: (−) represents 0 or <5% tumor cells; (+) represents 5–25% tumor cells; (++) represents 25–50% tumor cells; and (+++) represents the strongest staining with >50% tumor cells present. The immunoreactivity of cav-1 was normally localized to fibroblasts, type I pneumocytes and endothelial cells of blood vessels in all tissue specimens examined, which served as an internal quality control in the immunohistochemistry analysis. The same above protocol without the primary antibody was used as a negative control. High expression of cav-1 was artificially defined if ≥50% of the tumor cells (+++) showed granular staining at the cell membrane and in the cytoplasm.

### Statistical analysis

The difference in the level of expression of cav-1 mRNA and protein in TT and paired TF specimens was performed using the paired t-test. Associations between cav-1 protein expression and the clinicopathological characteristics were analyzed using the Mann-Whitney U-test. Overall survival (OS) was calculated from the day of surgery to the date of the last follow-up or the date of death. Survival at the last follow-up date was censored. The postoperative survival curves were calculated using the Kaplan-Meier method and differences in the survival rates were analyzed using the log-rank test. All statistical procedures were performed using SPSS (Version 16.0 SPSS Inc., Chicago). Values of P<0.05 were considered as statistically significant.

## Results

### Characteristics of the NSCLC cases

The 136 patients consisted of 100 males and 36 females, aged 17–80-years-old (mean 59.7-years-old). According to the classification of the World Health Organization (WHO), the specimens were classified into 74 (54.4%) AC, 44 (32.4%) SCC, and 18 (13.2%) others (large cell carcinomas, adenosquamous carcinomas and carcinoid). The average tumor size was 4.42±1.35 cm (range: 1–9 cm). Thirty-five cases involved tumors ≤3 cm in size, whereas 101 cases involved tumors >3 cm in size. There were 61 negative cases and 75 positive cases for lymph node metastases. The patient characteristics are presented in Tables I and II.

### Expression of caveolin-1 mRNA and protein in NSCLC paired tissues

**Figure 1:**
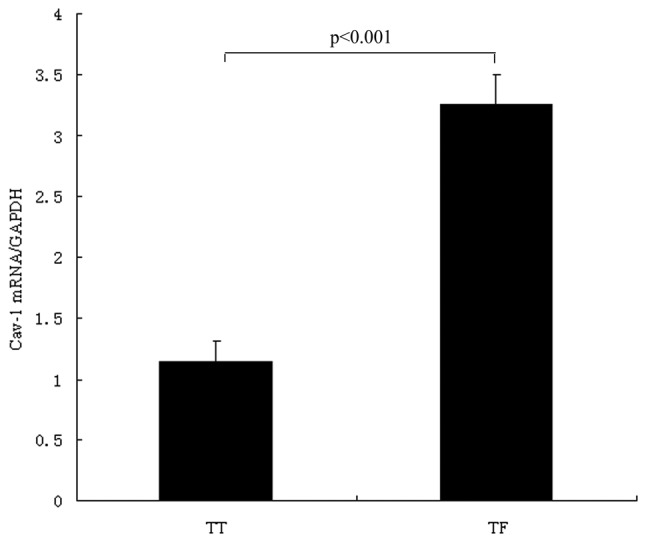
The relative level of cav-1 mRNA in 136 lung TT and TF samples (P<0.05).

We detected the mRNA levels of cav-1 in 136 paired specimens with lung TT and TF by SYBR-Green real-time PCR. The relative mRNA level of the cav-1 gene (cav-1 per GAPDH, mean ± SD) showed significant differences between lung cancer tissue (1.146±0.167) and the surrounding normal lung tissue (3.254±0.248). The paired t-test showed that cav-1 in TT was significantly lower than in TF (P<0.001) (Fig. 1). In summary, the cav-1 mRNA level is down-regulated in tumor samples of NSCLC.

**Figure 2:**
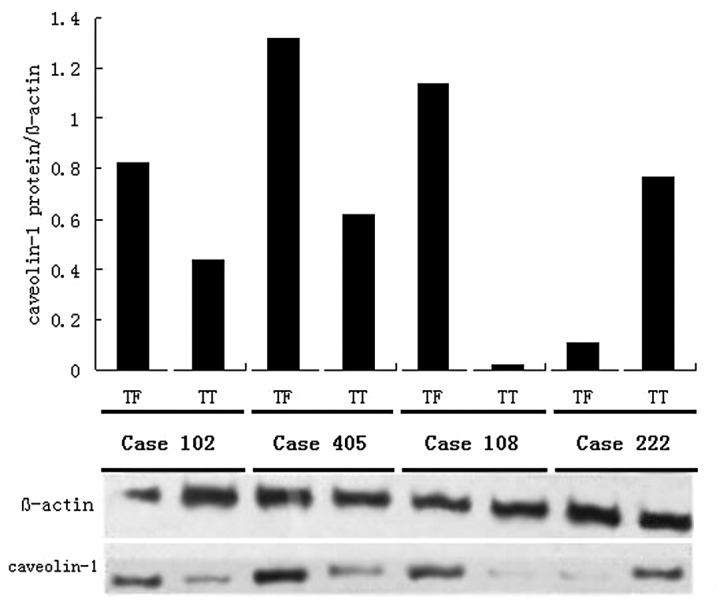
Western blot analysis of the protein level of cav-1 in TT and the paired TF form 4 representative NSCLC cases. β-actin was used to normalize for any differences in protein loading between lanes. The column of the histogram represents the relative level of the cav-1 protein.

In addition, 20 paired samples were analyzed by Western blot analysis. We confirmed that the protein expression of cav-1 was also down-regulated in TT. The protein level of cav-1 (cav-1 per β-actin, mean ± SD) was 0.56±0.39 in TT and 0.87±0.51 in TF, respectively. The paired t-test showed that cav-1 in TT was significantly lower than in TF (P=0.002), and 4 representative cases are shown in Fig. 2.

### Detection of caveolin-1 in NSCLC tissues and non-cancerous lung tissues

**Figure 3:**
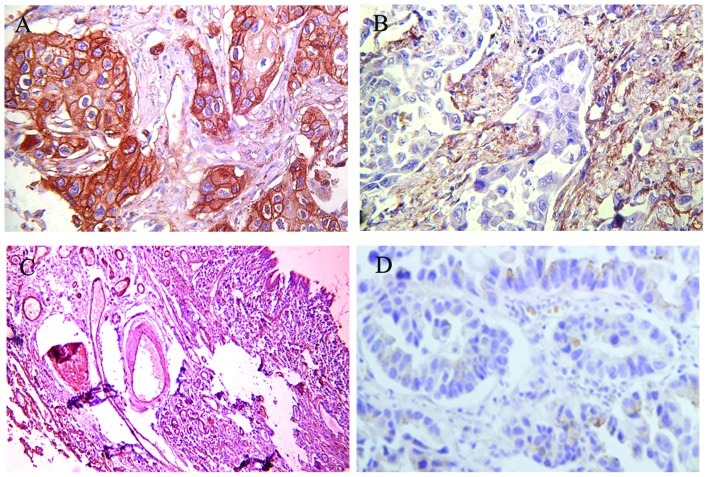
IHC of cav-1 protein expression in lung TT. (A) The positive immunoreactivity of cav-1 was localized in the membrane and the intracytoplasm of the lung AC. (B) The positive immunoreactivity of cav-1 was localized in fibroblasts. (C) Bronchial epithelium and smooth muscle cells showed positive staining for cav-1. (D) Negative expression of cav-1 in lung AC.

Immunohistochemistry was also performed to determine the expression and subcellular localization of the cav-1 protein in the 115 paraffin-embedded lung cancer specimens and 19 non-cancerous lung specimens. The positive immunoreactivity of cav-1 was localized in the membrane and the intracytoplasm of the lung AC (Fig. 3A). The majority of the cells in cancer or non-cancerous cases, including fibroblasts (Fig. 3B), type I pneumocytes, bronchial epithelium and smooth muscle cells (Fig. 3C), showed positive staining for cav-1. Negative expression of cav-1 is presented in Fig. 3D. Cav-1 overexpression was found in 60 of the 115 (52.2%) NSCLC patients, 23 of the 40 SCC (57.5%) and 32 cases of the 63 AC (50.8%). In addition, 15 of the 19 non-cancerous cases (78.9%) exhibited high expression levels of cav-1. Furthermore, the incidence of high cav-1 expression was significantly lower in NSCLC cases than non-cancerous cases (52.2% vs. 78.9%, P<0.05) (Table III).

### Relationship between the expression status of caveolin-1 mRNA and clinicopathological factors

To determine the significance of cav-1 mRNA expression in NSCLC, all patients were divided into 2 groups according to the relative expression levels (comparative to the TF). Analyzing these results in relation to the different histotypes, 22 of 74 (29.7%) lung AC, and 6 of 38 (15.8%) lung SCC showed up-regulated cav-1 mRNA expression and the difference by statistical analysis was significant (P=0.041). However, there was no significant correlation between the expression status of cav-1 mRNA and other clinicopathologic factors, such as gender, age, the size of the tumor, lymph node metastasis (pN) and p-TNM stages (P>0.05). The relationship between cav-1 mRNA expression and clinicopathologic factors is summarized in Table I.

### Relationship between the protein expression status of caveolin-1 and clinicopathological factors

We further evaluated the significance of cav-1 protein expression in NSCLC patients. The patients were divided into 2 groups according to the IHC evaluation criteria. The cav-1 protein expression levels and the association with clinicopathological variables are listed in Table II. There were no significant correlations observed between cav-1 expression and gender, age, histological type and the size of the tumor, pathological N-stage and pathological TNM-stage (P>0.05). We next performed a stratified analysis by histological type to evaluate the significance of the cav-1 protein expression in different histological types of NSCLC patients. Interestingly, a significant difference between high expression of cav-1 and poorer N-stage (P=0.032) and higher pathological TNM-stage (P=0.012) were only found in lung AC patients. However in lung SCC patients, there was no significant association between cav-1 protein expression and any other clinicopathological characteristics. Thus, the experimental data indicate that lung AC patients with high cav-1 protein expression levels show poorer N-stage and pathological TNM-stage.

### Survival analysis on caveolin-1 protein expression

**Figure 4:**
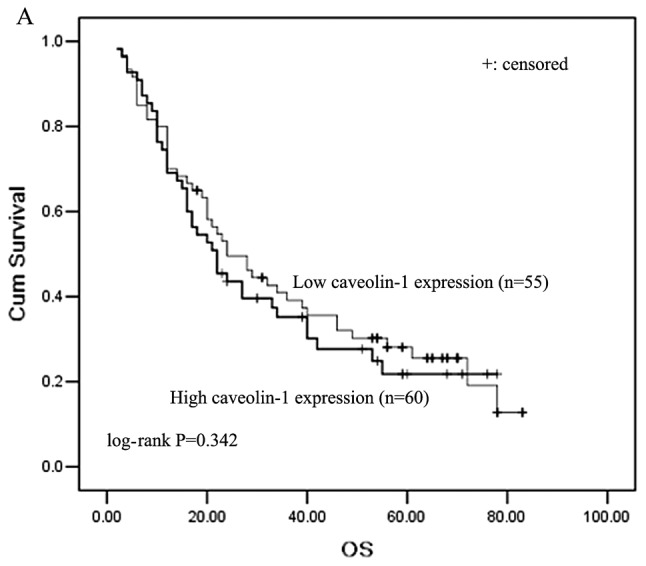

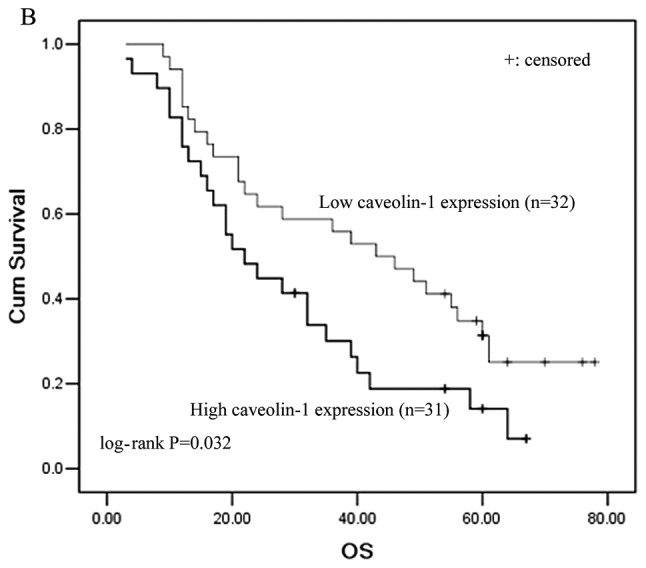
Survival analysis stratified by the status of the cav-1 protein expression and histological types. (A) Patients whose tumors had high cav-1 protein expression showed shorter overall survival than patients whose tumors had lower cav-1 protein expression, but the result was not statistically significant (P=0.342; log-rank test), (B) In all lung AC patients with higher cav-1 expression, significantly shorter OS was found when compared with those that had lower cav-1 expression levels (P=0.032; log-rank test).

In our study, there was inadequate clinical follow-up time for the NSCLC patients in which cav-1 mRNA was detected. Thus, we only analyzed the prognostic significance of cav-1 protein expression in the 115 paraffin-embedded NSCLC cases using IHC. The 5-year overall survival rate of all 115 patients was 41.3%. From Kaplan-Meier survival curves, we observed that patients with high cav-1 expression survived a shorter survival time than patients with low cav-1 levels (5-year survival rates, 22.3 and 29.2%, respectively); however, the result was not statistically significant (P=0.342, log-rank test, Fig. 4A). When stratified analysis by histological type was carried out, we found that lung AC patients with higher cav-1 expression showed significantly shorter survival than those with lower cav-1 expression, which indicated significantly poorer survival for higher cav-1 expression (P=0.032, log-rank test; Fig. 4B). However, in lung SCC patients, cav-1 was not a prognostic marker for overall survival.

## Discussion

This is the first study to extensively investigate the cav-1 mRNA and protein levels in paired TT and TF tissues, and their association with clinicopathological features. In this study, we showed that the levels of cav-1 mRNA and protein expression were significantly lower in lung TT than in matched TF tissue. Using IHC, the protein expression of cav-1 was significantly lower in NSCLC cases than in non-cancerous lung tissues. Up-regulation of cav-1 mRNA expression was found in lung AC more than lung SCC. We showed that the higher cav-1 protein expression closely correlated with poorer N-stage (P=0.032) and higher pathological TNM stage (P=0.012) in lung AC patients; however, no significant association between cav-1 protein expression and any other clinicopathological characteristics was found in lung SCC. Moreover, the most important point revealed in this study was that lung AC patients with higher cav-1 expression levels showed significantly shorter survival than those with lower cav-1 expression levels.

Cav-1, the principal structural protein in caveolae, has been recognized as a key player in the regulation of several signal transduction molecules, such as the HARS protein, epidermal growth factor (EGF) receptor, Src family tyrosine kinase, protein kinase C, transforming growth factor b/SMAD and the Wnt/β-catenin/lef-1 pathway (26–30). Cav-1 is an interesting molecule because of its apparent paradoxical biological functions in malignant tumors. Two previous studies showed that the expression of the cav-1 gene was down-regulated in lung cancer cells compared to normal bronchial epithelial cells. Decreased expression of cav-1 has also been found (9–10) in a variety of cancer cell lines of breast carcinoma, colon carcinoma, uterine cervical carcinoma and ovary (6–8,11). Conversely, in an *in vivo* study, overexpression of cav-1 was also observed in many human cancer tissues: bladder cancer (14), esophageal cancer (15), and prostate carcinomas (16). In our study, we used real-time PCR and Western blotting assays to detect the expression of cav-1 mRNA and protein in lung cancer tissues and matched TF tissues. The protein expression of cav-1 was significantly lower in NSCLC cases than in non-cancerous lung tissues. Our results indicate that the levels of cav-1 mRNA and protein expression were significantly decreased in lung TT when compared to matched TF tissue.

In total, 4 clinical studies (19–23) demonstrated the clinicopathological variables and the prognostic significance of cav-1 in resected NSCLC. Wikman and colleagues (23) investigated the cav-1 protein levels with IHC in all histological types of NSCLC and revealed that patients with high cav-1 showed no correlation with the disease outcome when all histological types were analyzed as 1 group or separately. In contrast, 3 other studies documented conflicting results. In two studies (20,21) the expression of cav-1 was associated with poor prognosis of patients with lung SCC and lung pleomorphic carcinoma. Ho *et al*, (19) revealed that up-regulated cav-1 could accentuate the metastasis capability of lung AD, and was an independent functional predictor of poor survival in lung AD. In our study, we showed that high protein expression of cav-1 was significantly correlated with poor survival in lung AC patients.

Cav-1 has been shown in *in vitro* studies to be down-regulated in various tumor cells including NSCLC (9,10). In our *in vivo* study, the results indicate that the levels of cav-1 mRNA and protein expression were significantly decreased in lung TT compared to matched TF tissue, and we confirmed the concept that cav-1 is down-regulated in NSCLC. Previous studies (31) have reported the cav-1 expression was present in alveolar epithelial type I (ATI) lung cells, but absent in its progenitor, the alveolar epithelial type II (ATII) cells. However, in the process of cell culturing, cav-1 expression increased with the signal at 192 h post-seeding being up to 50-fold greater than at 60 h. In addition, the study by Ho *et al*, (19) revealed that re-introduction of cav-1 expression into less invasive lung carcinoma cells caused an increase in cell invasive ability. These results, along with our observation that high expression of the cav-1 protein correlated with pN and poor over survival in lung AC patients, supports the concept that the function of cav-1 varies in the development and progression of lung cancer. In the deterioration and progression of lung cancer, cav-1 can be up-regulated, which may contribute to tumor invasion and metastasis.

In summary, we have shown that the levels of cav-1 mRNA and protein expression were significantly lower in lung TT than in matched TF tissue. Up-regulation of cav-1 mRNA expression was found in lung AC more than lung SCC. The higher cav-1 protein expression closely correlated with poorer N-stage and higher pathological TNM-stage in lung AC patients. Moreover, the most important point in this study was the observation that lung AC patients with higher cav-1 expression showed significantly shorter survival than those with lower cav-1 expression.

## Figures and Tables

**Table I:** Clinicopathological factors of lung cancer and the association with caveolin-1 mRNA expression in 136 NSCLC patients.

		Cav-1 mRNA expression		
		
	n (%)	Low	High	P-value
Number of patients	136	104	32	
Gender				0.656
Male	100 (73.5)	75	25	
Female	36 (26.5)	29	7	
Age				0.194
<60 years	65 (47.8)	46	19	
≥60 years	71 (52.2)	58	13	
Size of tumor				0.558
≤3 cm	35 (25.7)	25	10	
>3 cm	101 (74.3)	79	22	
Histology				0.041^a^
Squamous cell carcinoma	44 (32.4)	38	6	
Adenocarcinoma	74 (54.4)	52	22	
Other	18 (13.2)	14	4	
Lymph node metastasis (pN)				0.062
N0	61 (44.9)	51	10	
N1–3	75 (55.1)	53	22	
p-TNM stages				0.076
I	54 (39.7)	44	10	
II	27 (19.9)	19	8	
III	52 (38.2)	38	14	
IV	3 (2.2)	3	0	

a Statistically significant difference (P<0.05).

**Table II:** Clinicopathological characteristics of lung cancer and the association with caveolin-1 protein expression in 115 NSCLC patients.

		Caveolin-1 protein expression		
		
	n (%)	Low	High	P-value
Number of patients	115 (100)	55	60	
Gender				0.865
Male	87 (75.7)	42	45	
Female	28 (24.3)	13	15	
Age				0.452
<60 years	44 (38.3)	23	21	
≥60 years	71 (61.7)	32	39	
Smoking				0.187
Non-smoker	47 (40.9)	19	28	
Smoker	68 (59.1)	36	32	
Size of tumor				0.124
≤3 cm	32 (27.8)	19	13	
>3 cm	83 (72.2)	36	47	
Histology				0.596
Squamous cell carcinoma	40 (34.8)	17	23	
Adenocarcinoma	63 (54.8)	31	32	
Other	12 (10.4)	7	5	
N-stage				0.673
N0	42 (36.5)	19	23	
N1 + N2	73 (63.5)	36	37	
p-TNM stages				0.432
I	36 (31.3)	15	21	
II	27 (23.5)	11	16	
III	41 (35.7)	22	19	
IV	11 (9.6)	7	4	

**Table III:** Caveolin-1 expression in NSCLC and non-cancerous tissues.

		Caveolin-1		
			
	n	Low	High	P-value
NSCLC	115	55	60	0.029
Non-cancerous	19	4	15	
